# Supplementary material for: Multi‐dataset identification of innovative feature genes and molecular mechanisms in keratoconus
Source: J Cell Mol Med. 2024 Sep 19;28(18):e70079. doi: 10.1111/jcmm.70079 (PMC11412914; doi:10.1111/jcmm.70079)
Supplement: Supplementary file 2 — Material S2. [file JCMM-28-e70079-s002.zip › 7.GSEA analysis/5.RNF217-AS1/KEGG_VASCULAR_SMOOTH_MUSCLE_CONTRACTION.html]

Details for gene set KEGG\_VASCULAR\_SMOOTH\_MUSCLE\_CONTRACTION[GSEA]

|  || Dataset | RNF217-AS1.group.cls#h\_versus\_l |
| Phenotype | group.cls#h\_versus\_l |
| Upregulated in class | h |
| GeneSet | KEGG\_VASCULAR\_SMOOTH\_MUSCLE\_CONTRACTION |
| Enrichment Score (ES) | 0.21725853 |
| Normalized Enrichment Score (NES) | 0.77283716 |
| Nominal p-value | 0.83431953 |
| FDR q-value | 1.0 |
| FWER p-Value | 1.0 |
Table: GSEA Results Summary

  

Fig 1: Enrichment plot: KEGG\_VASCULAR\_SMOOTH\_MUSCLE\_CONTRACTION      
 Profile of the Running ES Score & Positions of GeneSet Members on the Rank Ordered List

  

| SYMBOL | TITLE | RANK IN GENE LIST | RANK METRIC SCORE | RUNNING ES | CORE ENRICHMENT || 1 | MYH11 | na | 61 | 0.810 | 0.0475 | Yes |
| 2 | PRKCG | na | 203 | 0.657 | 0.0834 | Yes |
| 3 | ADRA1D | na | 846 | 0.463 | 0.0930 | Yes |
| 4 | PTGIR | na | 1200 | 0.419 | 0.1082 | Yes |
| 5 | GUCY1A2 | na | 1454 | 0.397 | 0.1251 | Yes |
| 6 | CALML6 | na | 1514 | 0.391 | 0.1472 | Yes |
| 7 | ADCY2 | na | 2970 | 0.293 | 0.1230 | Yes |
| 8 | AGTR1 | na | 3697 | 0.263 | 0.1180 | Yes |
| 9 | PRKCQ | na | 3731 | 0.262 | 0.1330 | Yes |
| 10 | NPR2 | na | 4133 | 0.248 | 0.1365 | Yes |
| 11 | ADCY4 | na | 4384 | 0.238 | 0.1438 | Yes |
| 12 | NPR1 | na | 4698 | 0.228 | 0.1486 | Yes |
| 13 | ADCY5 | na | 4777 | 0.225 | 0.1600 | Yes |
| 14 | PLA2G4E | na | 4967 | 0.218 | 0.1678 | Yes |
| 15 | MYLK2 | na | 5508 | 0.199 | 0.1643 | Yes |
| 16 | BRAF | na | 5517 | 0.199 | 0.1762 | Yes |
| 17 | CACNA1F | na | 5544 | 0.198 | 0.1875 | Yes |
| 18 | ADCY7 | na | 5579 | 0.197 | 0.1984 | Yes |
| 19 | PLA2G2F | na | 5587 | 0.196 | 0.2101 | Yes |
| 20 | GNAQ | na | 7033 | 0.152 | 0.1777 | Yes |
| 21 | CACNA1S | na | 7170 | 0.148 | 0.1827 | Yes |
| 22 | PLCB3 | na | 7444 | 0.141 | 0.1834 | Yes |
| 23 | PLA2G2C | na | 7467 | 0.141 | 0.1914 | Yes |
| 24 | KCNMA1 | na | 7507 | 0.140 | 0.1987 | Yes |
| 25 | CACNA1C | na | 7536 | 0.139 | 0.2064 | Yes |
| 26 | ADRA1B | na | 7589 | 0.138 | 0.2133 | Yes |
| 27 | PLA2G2D | na | 7735 | 0.134 | 0.2173 | Yes |
| 28 | PRKG1 | na | 8062 | 0.126 | 0.2155 | No |
| 29 | PPP1R14A | na | 8688 | 0.112 | 0.2043 | No |
| 30 | ADORA2B | na | 8864 | 0.108 | 0.2058 | No |
| 31 | PPP1R12B | na | 8991 | 0.105 | 0.2086 | No |
| 32 | CACNA1D | na | 8999 | 0.105 | 0.2147 | No |
| 33 | ROCK1 | na | 10313 | 0.081 | 0.1817 | No |
| 34 | PLA2G12B | na | 10412 | 0.079 | 0.1837 | No |
| 35 | ADCY1 | na | 10624 | 0.076 | 0.1823 | No |
| 36 | JMJD7-PLA2G4B | na | 10713 | 0.074 | 0.1842 | No |
| 37 | KCNMB1 | na | 11416 | 0.064 | 0.1679 | No |
| 38 | ITPR3 | na | 12500 | 0.049 | 0.1395 | No |
| 39 | RAMP3 | na | 12616 | 0.047 | 0.1391 | No |
| 40 | RAMP2 | na | 13222 | 0.039 | 0.1240 | No |
| 41 | MAPK1 | na | 13362 | 0.038 | 0.1223 | No |
| 42 | CYP4A22 | na | 13537 | 0.036 | 0.1194 | No |
| 43 | CALM2 | na | 13884 | 0.032 | 0.1114 | No |
| 44 | MAPK3 | na | 14127 | 0.029 | 0.1062 | No |
| 45 | PLA2G10 | na | 14207 | 0.028 | 0.1056 | No |
| 46 | RHOA | na | 14935 | 0.020 | 0.0858 | No |
| 47 | PLA2G2E | na | 14946 | 0.020 | 0.0867 | No |
| 48 | CALML5 | na | 15459 | 0.014 | 0.0728 | No |
| 49 | PRKCD | na | 15568 | 0.013 | 0.0705 | No |
| 50 | MAP2K2 | na | 16236 | 0.006 | 0.0516 | No |
| 51 | PRKCH | na | 16241 | 0.006 | 0.0519 | No |
| 52 | GNA11 | na | 16472 | 0.004 | 0.0455 | No |
| 53 | PLA2G6 | na | 16529 | 0.003 | 0.0441 | No |
| 54 | PLA2G4A | na | 17031 | -0.002 | 0.0297 | No |
| 55 | CYP4A11 | na | 17137 | -0.003 | 0.0269 | No |
| 56 | GNA13 | na | 17152 | -0.003 | 0.0266 | No |
| 57 | ADCY9 | na | 17206 | -0.004 | 0.0253 | No |
| 58 | CALML3 | na | 17310 | -0.005 | 0.0226 | No |
| 59 | GNAS | na | 17807 | -0.009 | 0.0089 | No |
| 60 | PPP1CB | na | 18110 | -0.012 | 0.0009 | No |
| 61 | CALM3 | na | 18318 | -0.014 | -0.0043 | No |
| 62 | ADRA1A | na | 18508 | -0.016 | -0.0088 | No |
| 63 | MYL6 | na | 18616 | -0.017 | -0.0109 | No |
| 64 | ROCK2 | na | 18737 | -0.018 | -0.0133 | No |
| 65 | RAF1 | na | 18969 | -0.020 | -0.0187 | No |
| 66 | MYL6B | na | 19180 | -0.022 | -0.0235 | No |
| 67 | ADCY6 | na | 19355 | -0.024 | -0.0271 | No |
| 68 | MYLK | na | 19361 | -0.024 | -0.0258 | No |
| 69 | AVPR1B | na | 19611 | -0.026 | -0.0313 | No |
| 70 | PLA2G12A | na | 20757 | -0.037 | -0.0621 | No |
| 71 | GNA12 | na | 20990 | -0.040 | -0.0664 | No |
| 72 | PPP1CC | na | 21177 | -0.042 | -0.0693 | No |
| 73 | PLCB4 | na | 21374 | -0.044 | -0.0723 | No |
| 74 | ADCY8 | na | 21903 | -0.050 | -0.0845 | No |
| 75 | ITPR2 | na | 21984 | -0.051 | -0.0837 | No |
| 76 | PPP1CA | na | 22549 | -0.057 | -0.0966 | No |
| 77 | PPP1R12A | na | 22739 | -0.059 | -0.0985 | No |
| 78 | ADCY3 | na | 22794 | -0.059 | -0.0964 | No |
| 79 | PRKACA | na | 23066 | -0.063 | -0.1004 | No |
| 80 | MAP2K1 | na | 23215 | -0.064 | -0.1008 | No |
| 81 | ACTA2 | na | 23649 | -0.070 | -0.1090 | No |
| 82 | PRKCA | na | 23805 | -0.072 | -0.1091 | No |
| 83 | MYLK3 | na | 24189 | -0.078 | -0.1154 | No |
| 84 | ARAF | na | 25299 | -0.095 | -0.1416 | No |
| 85 | ACTG2 | na | 25398 | -0.097 | -0.1386 | No |
| 86 | ITPR1 | na | 25459 | -0.098 | -0.1344 | No |
| 87 | CALD1 | na | 26193 | -0.110 | -0.1488 | No |
| 88 | MYL9 | na | 26370 | -0.114 | -0.1470 | No |
| 89 | PRKACB | na | 26457 | -0.116 | -0.1424 | No |
| 90 | CALM1 | na | 26557 | -0.117 | -0.1382 | No |
| 91 | RAMP1 | na | 27671 | -0.140 | -0.1618 | No |
| 92 | ARHGEF1 | na | 27818 | -0.144 | -0.1572 | No |
| 93 | CALCRL | na | 27864 | -0.145 | -0.1497 | No |
| 94 | EDNRA | na | 28814 | -0.171 | -0.1667 | No |
| 95 | PLA2G1B | na | 29143 | -0.180 | -0.1652 | No |
| 96 | AVPR1A | na | 29821 | -0.201 | -0.1726 | No |
| 97 | ADORA2A | na | 30043 | -0.208 | -0.1663 | No |
| 98 | PLA2G5 | na | 30416 | -0.220 | -0.1636 | No |
| 99 | KCNMB3 | na | 30642 | -0.228 | -0.1562 | No |
| 100 | PLCB2 | na | 31149 | -0.248 | -0.1558 | No |
| 101 | PRKX | na | 31362 | -0.257 | -0.1463 | No |
| 102 | PRKCE | na | 31530 | -0.265 | -0.1350 | No |
| 103 | ARHGEF11 | na | 31685 | -0.273 | -0.1228 | No |
| 104 | PLCB1 | na | 32371 | -0.315 | -0.1235 | No |
| 105 | PLA2G2A | na | 33013 | -0.361 | -0.1200 | No |
| 106 | PRKACG | na | 33174 | -0.374 | -0.1019 | No |
| 107 | KCNMB2 | na | 33406 | -0.396 | -0.0845 | No |
| 108 | PLA2G3 | na | 33574 | -0.415 | -0.0641 | No |
| 109 | ARHGEF12 | na | 33601 | -0.419 | -0.0393 | No |
| 110 | PRKCB | na | 34175 | -0.520 | -0.0242 | No |
| 111 | KCNMB4 | na | 34511 | -0.661 | 0.0063 | No |
Table: GSEA details [plain text format]

  

Fig 2: KEGG\_VASCULAR\_SMOOTH\_MUSCLE\_CONTRACTION      
 Blue-Pink O' Gram in the Space of the Analyzed GeneSet

  

Fig 3: KEGG\_VASCULAR\_SMOOTH\_MUSCLE\_CONTRACTION: Random ES distribution      
 Gene set null distribution of ES for **KEGG\_VASCULAR\_SMOOTH\_MUSCLE\_CONTRACTION**
